# Study on Sensing Mechanism of Volatile Organic Compounds Using Pt-Loaded ZnO Nanocrystals

**DOI:** 10.3390/s22166277

**Published:** 2022-08-20

**Authors:** Takeshi Shinkai, Keigo Masumoto, Masaru Iwai, Yusuke Inomata, Tetsuya Kida

**Affiliations:** 1 Department of Material Science and Applied Chemistry, Graduate School of Science and Technology, Kumamoto University, Kumamoto 860-8555, Japan; 2 Division of Materials Science, Faculty of Advanced Science and Technology, Kumamoto University, Kumamoto 860-8555, Japan; 3 Institute of Industrial Nanomaterials, Kumamoto University, Kumamoto 860-8555, Japan; 4 International Research Organization for Advanced Science and Technology (IROAST), Kumamoto University, Kumamoto 860-8555, Japan

**Keywords:** semiconductor gas sensor, Pt-loading, ZnO, ethanol, DRIFT, catalytic activity

## Abstract

Understanding the surface chemistry of target gases on sensing materials is essential for designing high-performance gas sensors. Here, we report the effect of Pt-loading on the sensing of volatile organic compounds (VOCs) with ZnO gas sensors, demonstrated by diffuse reflection infrared Fourier transform (DRIFT) spectroscopy. Pt-loaded ZnO nanocrystals (NCs) of 13~22 nm are synthesized using the hot soap method. The synthesized powder is deposited on an alumina substrate by screen-printing to form a particulate gas sensing film. The 0.1 wt% Pt-loaded ZnO NC sensor shows the highest sensor response to acetone and ethanol at 350 °C, while the responses to CO and H_2_ are small and exhibit good selectivity to VOCs. The gas sensing mechanism of ethanol with Pt-ZnO NCs was studied by in situ DRIFT spectroscopy combined with online FT-IR gas analysis. The results show that ethanol reacts with small Pt-loaded ZnO to produce intermediate species such as acetaldehyde, acetate, and carbonate, which generates a high sensor response to ethanol in air.

## 1. Introduction

Semiconductor gas sensors have been used in a wide range of fields, including safety monitoring, industrial disaster prevention, automotive exhaust control, and medical diagnosis, since the first report by Seiyama et al. in 1962 [1]. This type of sensor is finding widespread applications in a variety of fields because of its high sensitivity, robustness, simplicity, and low cost. The sensor device consists of metal oxide chemiresistors whose electrical resistance changes in response to gas adsorption and surface reactions. Typical materials include SnO_2_, ZnO, In_2_O_3_, and WO_3_ [1,2,3,4,5]. The well-accepted gas sensing mechanism is as follows. At high temperatures, oxygen adsorbs onto the surface of metal oxides, capturing free electrons and forming an electron depletion layer on the surface. This induces a high electrical resistivity state in the n-type oxide semiconductor. The captured electrons are released back into the original host through a reaction between the gas species and the adsorbed oxygen (combustion reaction). As a result, a low-resistivity state with a thinner depletion layer is achieved. The change in electrical resistance associated with this surface reaction is used as a sensor signal.

The above surface reaction is influenced by many internal and external factors, such as particle size, sensing layer microstructure, surface conditions, humidity, and temperature. One effective means of improving the sensor response is particle size control, which is referred to as the particle size effect [6,7]. If the size of the constituent particles is less than twice the thickness of the electron depletion layer, the entire particle becomes electron depleted due to oxygen adsorption. In this case, not only the particle surface, but also the bulk contributes to the change in electrical resistance during the surface reaction [6,8,9]. Therefore, reducing the particle size to less than approximately 10 nm often improves the sensor response. Decorating the surface of metal oxides with foreign additives such as noble metals has been widely implemented to upgrade the sensor performance [10,11,12,13]. Two sensitization mechanisms, i.e., chemical sensitization and electronic sensitization, were proposed by Yamazoe et al. in 1983 [10]. In the chemical sensitization, gas molecules dissociatively adsorb onto the additive surface and then migrate to the oxide surface via a spillover process. They react with the adsorbed oxygen on the oxide surface and increase the electrical conductivity of the oxide. In the electronic sensitization, additives are believed to interact electronically with the metal oxide to form p-n junctions or Schottky junctions at the interface. The reaction between the gas molecules and the additive changes the height of the potential barrier at the junction, resulting in a significant change in electrical resistivity. Thus, the two concepts, particle size control and surface modification, are key to high-performance gas sensors.

ZnO has been one of the useful gas sensing materials since its first report [2]. ZnO has found versatile applications to date, including solar cells [14,15], light-emitting diodes [16,17], and liquid crystal display [18,19] due to its promising electrical and optical properties. The gas sensing characteristics of ZnO have been well studied [20,21,22]. Its sensor response can be enhanced by coupling with novel metals [23,24] or metal oxides [25,26]. Control of the shape and size of ZnO is also effective in enhancing sensing performance [27,28]. Although ZnO-based sensors are widely used, their gas detection mechanism has not yet been fully elucidated. In particular, the interaction between the gas species and the ZnO surface needs to be investigated in more detail to clarify the correlation between the sensor response and surface reactivity. Diffuse reflection infrared Fourier transform (DRIFT) spectroscopy is a powerful method for analyzing the reaction of gaseous species on a solid surface [29,30,31]. Staerz et al. used DRIFT spectroscopy to study the surface reactions of WO_3_ sensors with different test gases, such as NO_2_ and CO [5]. Boehme et al. revealed a mechanism of CO sensing in the presence of humidity in In_2_O_3_ sensors with the DRIFT technique [32].

In this study, to understand the role of surface chemistry in gas sensing, the surface reactivity of volatile organic compound (VOC) gases on ZnO was studied using DRIFT spectroscopy. We focused on the sensor response of ZnO NCs synthesized by a hot soap method [33]. These NCs are approximately 10–20 nm in size and have a narrow size distribution. Highly crystalline and homogeneous samples are suitable for characterizing the sensing properties of ZnO nanoparticles. Gas sensing measurements were conducted using six different gases, including H_2_, CO, ethanol, acetone, and toluene, under dry conditions. The effect of Pt-loading on the sensor response was also investigated. The surface reaction of ethanol on ZnO NCs was analyzed by in situ DRIFTS measurements with on-line Fourier transform infrared spectroscopy (FT-IR) gas analysis.

## 2. Materials and Methods

### 2.1. Synthesis of Pt-Loaded ZnO NCs

Pt-loaded ZnO nanocrystals (NCs) were synthesized by the hot soap method [33]. 1 mmol of Zn(II) acetylacetonate (Zn(C_5_H_7_O_2_)_2_·xH_2_O) (purity 98%, Wako Pure Chemical Industries, Osaka, Japan), 10 mmol of 1,2-hexadecanediol (CH_3_(CH_2_)_13_CHOHCH_2_OH) (purity 90%, St. Louis, MO, USA), 5 mL of oleic acid (C_17_H_33_COOH) (purity 65%, Wako Pure Chemical Industries, Osaka, Japan), and 15 mL of oleylamine (C_18_H_37_N) (purity 100%, Wako Pure Chemical Industries, Osaka, Japan) were loaded into a three-neck flask, and connected to a Schlenk line. The system was heated at 80 °C for 30 min under a vacuum. Then, the temperature was increased to 220 °C under an Ar flow, and the reaction was carried out for 1.5 h. The synthesized ZnO NCs were recovered via centrifugation at 8000 rpm for 10 min. The precipitates were washed with hexane three times.

Pt nanoparticles were deposited on the ZnO NCs. Platinum–Oleylamine (Pt-OA) complex was synthesized as follows. In a glove box, 0.602 g of hydrogen hexachloroplatinate (IV) hexahydrate (H_2_PtCl_6_·6H_2_O) (purity 98.5%, Wako Pure Chemical Industries, Osaka, Japan) and 15.2 mL of oleylamine (OA: C_18_H_37_N) (purity 100%, Wako Pure Chemical Industries, Osaka, Japan) were loaded into a three-necked flask and heated at 40 °C for 2 h under vacuum with stirring. A designated amount of the solution containing the Pt-OA complex was added to the precursor solution used for the synthesis of ZnO NCs. The amounts of the Pt-complex solution added were 8, 40, 80, and 400 μL, which correspond to 0.1, 0.5, 1, and 5 wt% of Pt loading, respectively. The synthesis of Pt-ZnO NCs was carried out at 220 °C in Ar for 2 h. The synthesized samples are denoted as xPt/ZnO NCs (x = 0, 0.1, 0.5, 1, 5), respectively.

### 2.2. Materials Characterization

The crystal structures of all synthesized materials were characterized by X-ray diffraction (XRD) (MiniFlex600; Rigaku, Tokyo, Japan) using a monochromatic Cu-Kα radiation source (λ = 1.541862Å). XRD patterns were recorded from 20 to 80° with a step size of 0.02° at the scan rate of 10 °/min. Transmission electron microscopy (TEM) (JEM-200FX, JEOL, Tokyo, Japan), high-angle annular dark-field scanning transmission electron microscopy (HAADF-STEM) (Tecnai F20, Thermo Fisher Scientific, Waltham, MA, USA), and field emission scanning electron microscopy (FE-SEM) (JSM 7600 F, JEOL, Tokyo, Japan) were used for morphological analysis of the samples. The loading amounts of Pt on ZnO were determined by X-ray fluorescence (XRF) analysis (Epsilon 1, Malvern Panalytical, Malvern, UK).

### 2.3. Fabrication of Gas Sensor Devices

The synthesized samples were grounded and mixed with α-terpineol to obtain a homogeneous paste. Using a commercial screen-printing machine (MEC-2400E, Mitani Micronics Co., Ltd., Saga, Japan), the paste was screen-printed through a screen mesh (8 × 8 mm^2^) on an alumina substrate (9 × 13 × 1 mm^3^) with gold combo-shaped electrodes to form a gas sensing layer. Alumina substrates were treated with an aqueous solution containing ammonia (NH_3_) (assay 28–30%, Wako Pure Chemical Industries, Osaka, Japan) and hydrogen peroxide (H_2_O_2_)) (assay 30–35.5%, Wako Pure Chemical Industries, Osaka, Japan) at 80 °C for 10 min and then treated by a UV ozone cleaner (UV253-OZ, Filgen Co., Ltd., Aichi, Japan) before screen-printing. The sensor device was annealed at 450 °C for 10 min in an electric furnace (KDF-S70, Denken Co., Ltd., Oita, Japan) to remove surface ligands from the NC surface.

### 2.4. Gas Sensing Measurements

**Figure 1:**
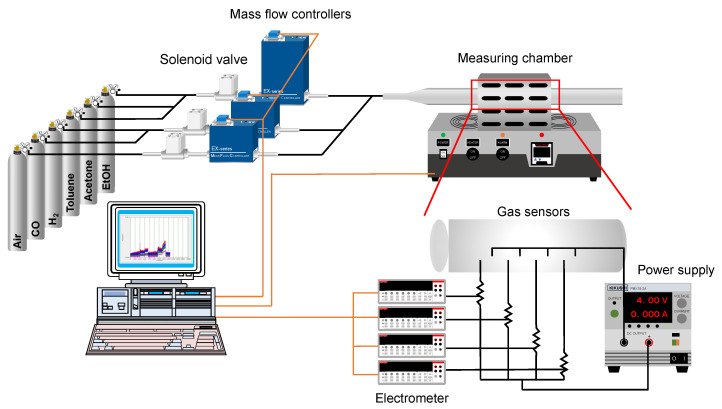
Schematics of the experimental setup for gas sensing measurements.

Figure 1 shows a schematic diagram of the experimental setup, which allows the performance of four sensor devices to be evaluated simultaneously. The sensor devices were loaded into a gas flow chamber that was externally heated by an electric furnace (FT-02VAC-15, FULL-TECH Co., Ltd., Osaka, Japan). The test gases in synthetic air were introduced to the gas flow chamber. Standard gases H_2_ (1000 ppm), CO (1000 ppm), toluene (50 ppm), and acetone (50 ppm) were purchased from KUMAMOTO OXYGEN Co., Ltd. (Kyoto, Japan). Ethanol (50 ppm) was purchased from FUKUOKA OXYGEN Co., Ltd. (Nagasaki, Japan). The concentration of standard gases was adjusted by a lab-made, computer-controlled gas mixing system consisting of mass flow controllers, solenoid valves, and flow sensors. The flow rate of the mixed gas was set at 100 mL/min. The sensor device and a standard resistor were connected in series, and 4 V was applied to the circuit using a DC power supply (PMX18-2A, KIKUSUI, Kanagawa, Japan). The voltage applied to the standard resistor was measured with a multimeter (2100 USB Digital Multimeter, Tektronix, Inc., Beaverton, OR, USA). The electrical resistance values of the sensors were calculated using Equation (1). The sensor response (sensitivity) was defined in Equation (2). Where *R*_air_ is the resistance of the sensor in air, *R*_test gas_ is the electric resistance of the sensor in the test gas, *r* is the resistance of the standard resistor, and *V* is the voltage applied to the standard resistor.

(1) $$R=r\left(\frac{4}{V}-1\right)$$

(2) $$S=\frac{R_{\mathrm{air}}}{R_{\mathrm{test}\;\mathrm{gas}}}$$

### 2.5. In Situ DRIFT Spectroscopy with on-Line FT-IR Gas Analysis

For DRIFT spectroscopy, an FT-IR spectrometer (Nicolet iS50, Thermo Fisher Scientific, Waltham, MA, USA) with a spectral resolution of 4 cm^-1^ and a diffuse reflection accessory were used. The sensor device was loaded into a gas flow DRIFT chamber equipped with a heating plate and BaF_2_ windows (ST Japan, Tokyo, Japan). An incident IR beam diffused within a sensing layer made of ZnO NCs was reflected into a mercury cadmium telluride (MCT) detector through BaF_2_ windows. The sensor was heated at 350 °C in air or air containing 50 ppm ethanol. FT-IR absorbance spectra were recorded every 5 min.

Gaseous products formed by the combustion of ethanol on the sensor device were detected using online FT-IR gas analysis. Exhaust gas from the DRIFT chamber was introduced to a gas cell (path length: 2.4 m) (PIKE Technologies, Fitchburg, WI, USA) and was then analyzed with an FT-IR spectrometer (FT/IR-4100, JASCO, Tokyo, Japan).

## 3. Results and Discussion

### 3.1. Materials Characterization

**Figure 2:**
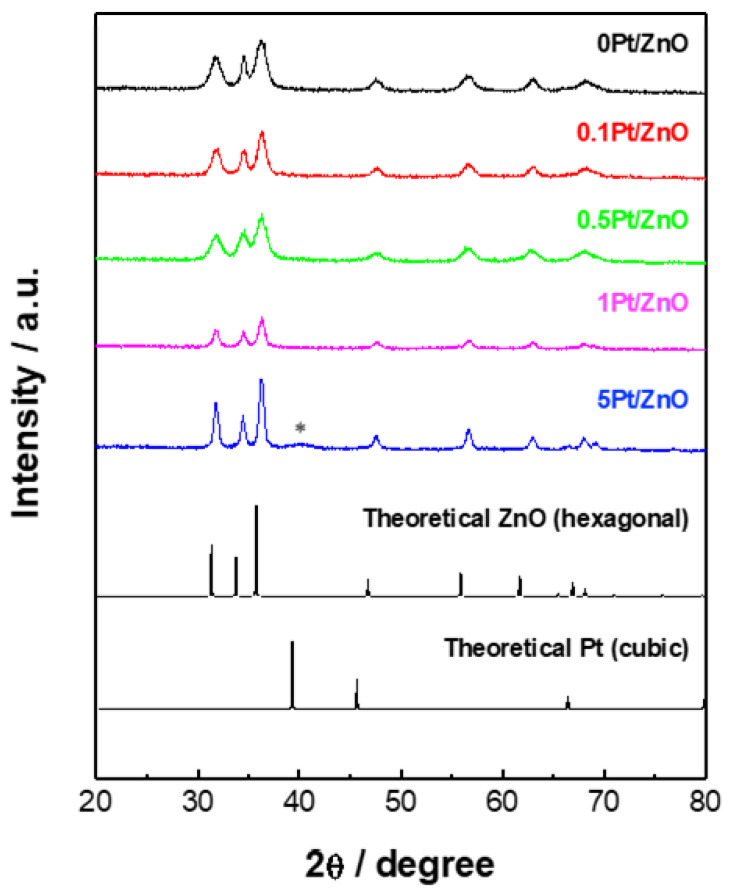
XRD patterns of ZnO NCs with different Pt-loading amounts. * Peak ascribed to Pt^0^.

The XRD patterns of all samples shown in Figure 2 are in good agreement with the theoretical pattern of hexagonal ZnO. The peaks of 5Pt/ZnO were broader than those of the other samples, suggesting that the crystal growth of ZnO NCs occurred in the presence of a larger amount of the Pt precursor. A peak attributed to the cubic Pt was observed at approximately 40° only in 5Pt/ZnO (marked with an asterisk). The size of ZnO NCs was examined by TEM analysis (Figure S1). The particle size of 0Pt/ZnO, 0.1Pt/ZnO, 0.5Pt/ZnO, 1Pt/ZnO, and 5Pt/ZnO is 13.8, 16.5, 13.1, 18.9, and 22.9 nm, respectively. The TEM images showed the presence of Pt NCs of 3.8~5.9 nm in 0.5Pt/ZnO, 1Pt/ZnO, and 5Pt/ZnO. They were not visible in 0.1Pt/ZnO due to the small loading amount. The colloidal size of ZnO NCs was analyzed by dynamic light scattering (DLS), as shown in Figure S2. The colloidal size increased with the increase in Pt loading, in good accordance with the TEM results.

**Figure 3:**
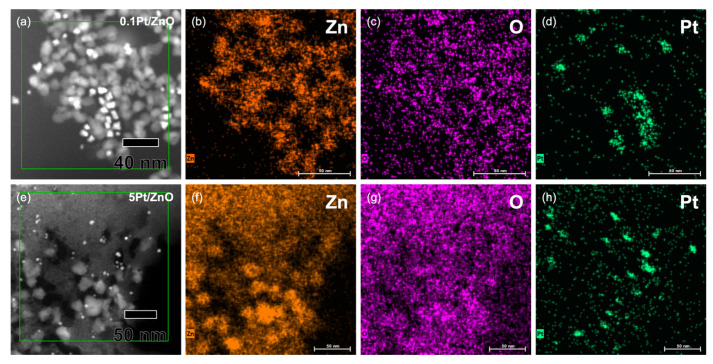
HAADF-STEM image of (**a**) 0.1Pt/ZnO and (**e**) 5Pt/ZnO NCs. EDS elemental mapping of (**b**–**d**) 0.1Pt/ZnO and (**f**–**h**) 5Pt/ZnO NCs. Green areas in (**a**,**e**) were magnified and analyzed by EDX.

The distribution of Pt on ZnO NCs was examined by high-angle annular dark-field scanning transmission electron microscopy (HAADF-STEM) and energy-dispersive X-ray (EDX) mapping, as shown in Figure 3. The images of 0.1Pt/ZnO and 5Pt/ZnO clearly show that Pt NCs were distributed on ZnO NCs. The size of Pt deposited on 0.1Pt/ZnO is 7.3 nm, which is larger than that of 5Pt/ZnO (4.4 nm). EDX spot spectra confirm the presence of Pt in 0.1Pt/ZnO and 5Pt/ZnO (Figure S3). The amount of Pt loading on ZnO determined by XRF analysis is shown in Table S1: the determined amounts of 0.1Pt/ZnO and 0.5Pt/ZnO are higher than the starting weight ratios of Pt/Zn, while those of 1Pt/ZnO and 5Pt/ZnO are lower. Pt NCs were not uniformly distributed on ZnO NCs; the aggregation of Pt was seen in the HAADF-STEM images. The noticeable phenomenon in the Pt-ZnO NC system is the further growth of ZnO NCs when increasing the amount of Pt. In the present synthesis method, the Zn and Pt precursors were heated together to 220 °C. The nucleation temperature of Pt^0^ should be lower than that of ZnO. Therefore, the earlier formed Pt NCs may have assisted the growth of ZnO NCs. This hypothesis is supported by the fact that the shape of ZnO NCs changed from spherical to irregular as the Pt content increased, as shown in Figure S1. The detailed mechanism, however, remains to be clarified.

**Figure 4:**
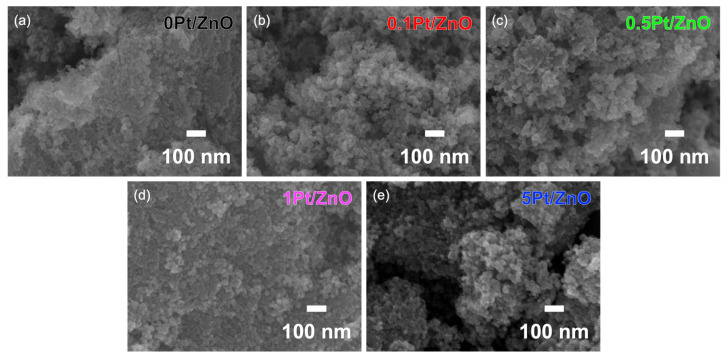
FE-SEM images of gas sensing films fabricated using (**a**) 0Pt/ZnO; (**b**) 0.1Pt/ZnO; (**c**) 0.5Pt/ZnO; (**d**) 1Pt/ZnO; (**e**) 5Pt/ZnO NCs.

The morphology of the sensing films composed of Pt-ZnO NCs was characterized by field emission scanning electron microscopy (FE-SEM), as shown in Figure 4. Densely packed particulate films were formed due to the uniform size of Pt-ZnO NCs. No significant crystal growth and sintering were seen. The images show that all films have 3 to 10 nm mesopores, indicating that gas diffusion in the films is possible [34]. The thickness of the sensing layers composed of 0Pt/ZnO, 0.1Pt/ZnO, and 5Pt/ZnO is 13.5, 18.2, and 17.9 μm, respectively (Figure S4).

### 3.2. Gas Sensing Properties

**Figure 5:**
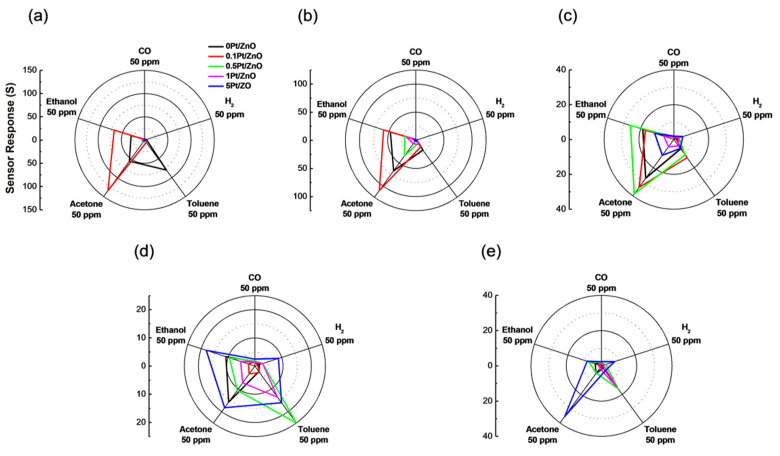
Sensor responses of Pt-ZnO NCs to five test gases at (**a**) 400; (**b**) 350; (**c**) 300; (**d**) 250; (**e**) 200 °C under dry conditions.

**Figure 6:**
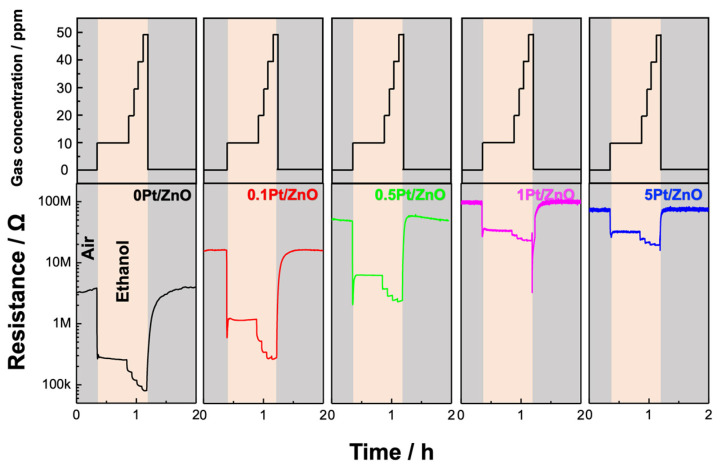
Response transients to ethanol at 350 °C under dry conditions for the device using 0, 0.1, 0.5, 1, and 5% Pt-loaded ZnO NCs. The upper panel indicates the gas concentration during the measurements.

Figure 5 shows the sensor response to five test gases in dry conditions at 400, 350, 300, 250, and 200 °C for the ZnO NCs with different Pt loadings. All Pt-ZnO NC sensors responded selectively to volatile organic compounds (VOCs), while the sensor responses to H_2_ and CO were small in all cases. The response speed was also good, with a rapid decrease in electrical resistance dependent on the concentration of each test gas, as shown in Figure 6. The resistance recovered quickly to the original value when test gases were switched to air. The dependence of the sensor response to the five different gases on temperature is shown in Figure S5. We found that 0.1Pt/ZnO NCs showed the highest sensitivity to acetone and ethanol at 350 and 400 °C, demonstrating the improvement in the gas sensing properties as a result of Pt loading.

The sensitivity of 0.1Pt/ZnO NCs decreased rapidly below 300 °C. Conversely, the sensor response of 0.5Pt/ZnO and 5Pt/ZnO NCs to ethanol was maximal at 300 and 250 °C, respectively. Another notable trend is the improved response of 0.5Pt/ZnO NCs to acetone and ethanol at 300 °C. 0.5Pt/ZnO and 1Pt/ZnO NCs showed a selective response to toluene at 250 °C, while 5Pt/ZnO was able to detect acetone at 200 °C, selectively.

Considering the previously reported effects of Pt loading on gas sensing properties [10,35,36], it is assumed that the test gas is completely oxidized (complete combustion) near the sensing film surface due to too strong catalytic effects of large amounts of Pt at high temperatures, making it difficult for the test gases to diffuse deep inside the film. For this reason, no significant change in electrical resistance appeared at 350 °C and 400 °C for the high Pt-loaded samples (0.5, 1, and 5Pt/ZnO). In contrast, 0.1Pt/ZnO showed the best sensor response at 350 °C. This is due to the moderate activation of VOC reaction by Pt in the sensing film at this temperature. On the other hand, at lower temperatures, VOCs may diffuse into the sensing film even with high Pt loading, and VOC gases avoid complete combustion at the film surface at 250 and 200 °C. The high Pt loading should promote the reaction between VOCs and ZnO deep inside the film and increase the sensor response at lower temperatures. It is speculated that further increasing the NC size will increase the mesopore size of the sensing film, allowing VOCs to diffuse more efficiently and penetrate deeper into the film, thereby improving gas sensitivity [37].

### 3.3. In Situ DRIFT Spectroscopy with on-Line FT-IR Gas Analysis

The combustion reaction of organic gases is particularly complex. Ochoa et al. reported that in the combustion reaction of ethanol on spinel mixed oxides, acetaldehyde is first formed by dehydrogenation of ethanol [38]. Acetaldehyde is then oxidized to acetate, which is either decomposed to methane and carbon monoxide, or completely oxidized to CO_2_. These intermediates, including acetaldehyde, acetate, and carbonate, likely form and adsorb on Pt-ZnO NCs. Such complex oxidation reaction pathways are expected to be involved the sensing mechanism. Therefore, in situ DRIFT spectroscopy was used to analyze the reactivity of VOCs with Pt-ZnO NCs. Here, we focused on ethanol reaction on ZnO, 0.1Pt/ZnO, and 5Pt/ZnO NCs. Ethanol reactions on Co/ZnO, Pt/Al_2_O_3_, Pt/CeO_2_, and Co/ZrO_2_ were also studied using DRIFT spectroscopy [38,39,40,41].

**Figure 7:**
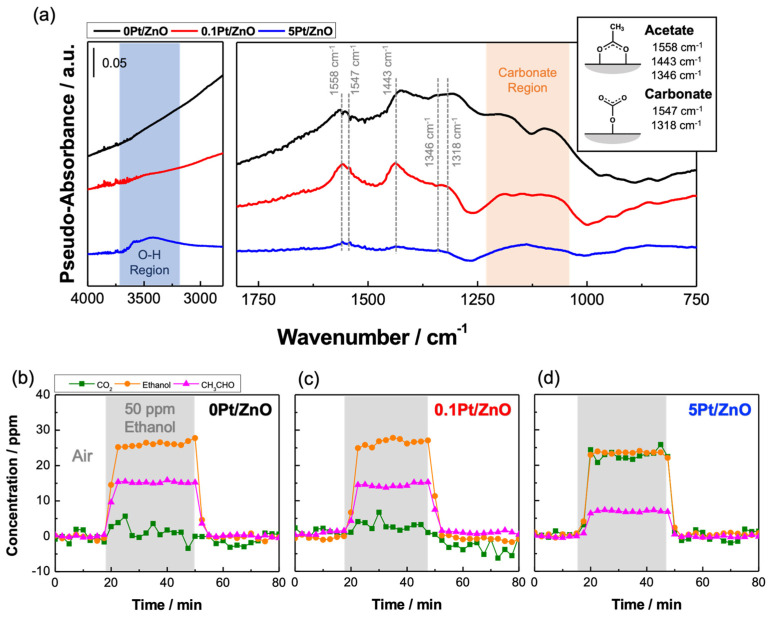
(**a**) DRIFT spectra of 0Pt/ZnO, 0.1Pt/ZnO, and 5Pt/ZnO NCs recorded after exposure to 50 ppm ethanol in dry synthetic air at 350 °C. Time dependence of the concentration of gaseous products formed by the combustion of ethanol on (**b**) 0Pt/ZnO, (**c**) 0.1Pt/ZnO, and (**d**) 5Pt/ZnO NCs at 350 °C.

Figure 7a shows the DRIFT spectra recorded after the three different samples were exposed to 50 ppm ethanol in the air at 350 °C for 30 min in dry conditions. 5Pt/ZnO showed a peak at around 3500 cm^−1^ due to the formation of hydroxyl groups. This peak can be attributed to adsorbed H_2_O generated by the combustion of ethanol. The results indicate that complete combustion of ethanol occurred on the 5Pt/ZnO surface due to the high amount of Pt. On the other hand, no OH groups were observed on ZnO and 0.1Pt/ZnO NCs, suggesting their catalytic activity for the complete combustion is moderate.

The DRIFT spectra showed adsorption of acetate (CH_3_COO) species at 1558, 1443, and 1346 cm^−1^. The peaks at 1547 and 1318 cm^−1^ and the broad band at 1200–1100 cm^−1^ also suggest the presence of carbonate (CO_3_) species. It has been reported that the peaks assigned to carbonate species vary depending on the adsorption form [41]. Thus, the peaks appeared as the band, as shown by the orange region in Figure 7a. These peaks and bands ascribed to acetate and carbonate were the most intense for 0.1Pt/ZnO, but were weaker for the highly Pt-loaded sample (5Pt/ZnO) at 350 °C. It is accepted that ethanol dissociatively adsorbs on the oxide surface to produce ethoxide (CH_3_CH_2_O) species, giving IR absorption at 2970, 2930, 2875, 1107, and 1065 cm^−1^ [42]. The ethoxide species are readily oxidized to form acetate species, giving IR absorption at 1558, 1443, and 1346 cm^−1^ [42]. Acetate species are reported to be further oxidized to carbonate species (1547, 1318 cm^−1^) [38]. However, in the present case, no peaks attributable to the ethoxide species appeared, suggesting that they do not react with ZnO or were quickly oxidized to acetate. In addition, the adsorption of acetaldehyde (CH_3_CHO) could not be observed.

FT-IR analysis of the exhaust gas was performed to corroborate the above explanation. Figure 7b,c shows that the exhaust gas that passed through the sensing layer consisting of 0Pt/ZnO and 0.1Pt/ZnO NCs contained approximately 27 ppm ethanol, 15 ppm acetaldehyde, and a few ppm CO_2_. The results indicate that ethanol was partially oxidized into acetaldehyde on 0Pt/ZnO and 0.1Pt/ZnO at 350 °C. Other products may also be produced, some of which may remain adsorbed on the ZnO NCs. On the other hand, for 5Pt/ZnO, a larger amount of CO_2_ was detected (Figure 7d). The highly loaded Pt accelerated the complete combustion of ethanol.

**Figure 8:**
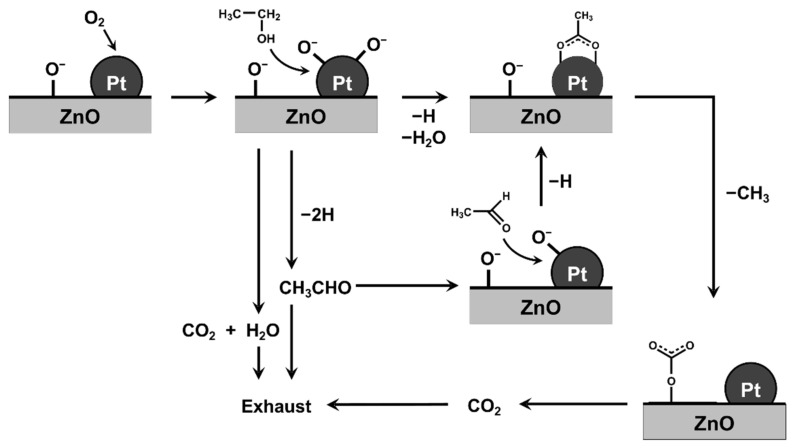
The schematic diagram of the combustion reaction of ethanol on the Pt-loaded ZnO surface.

Combining the DRIFT and FT-IR gas analysis results, we propose the reaction model shown in Figure 8. In the Pt-ZnO NC system, ethanol is oxidized to produce acetaldehyde or CO_2_ as gaseous products. The acetaldehyde re-adsorbed or remaining on the Pt-ZnO NC surface is rapidly converted into acetate by adsorbed oxygen, which is further oxidized into carbonate. The adsorption of intermediate species on Pt is thought to promote this conversion, resulting in the higher sensor response of 0.1Pt/ZnO NCs at 350 °C. The direct conversion of ethanol into acetate is also probable on Pt-ZnO NCs. On the other hand, in 5Pt/ZnO, the complete oxidation process is predominant, and there are fewer intermediate species on the NCs. Thus, in the sensing layer with high Pt loading, the reaction between ethanol and ZnO deep in the layer is avoided, resulting in a smaller change in electrical resistance at 350 °C. However, the critical step that determines the sensor response is still unknown. More detailed studies are currently underway.

## 4. Conclusions

Pt (0.1, 0.5, 1, and 5 wt%)-loaded ZnO NCs were synthesized using the hot soap method. The synthesized powder was mixed with α-terpineol to form a paste and deposited on an alumina substrate with Au electrodes via screen-printing. The results of TEM, HAADF-STEM, and EDS elemental mapping revealed that Pt nanoparticles (4.4–7.3 nm) are distributed on ZnO NCs (13–22 nm). 0.1Pt/ZnO NCs showed the highest sensor response to 50 ppm acetone and ethanol at 350 °C, exhibiting the promotion effect of Pt. The sensor response to ethanol of 0.5Pt/ZnO, 1Pt/ZnO, and 5Pt/ZnO NCs was maximal at 300, 350, and 250 °C, respectively. The surface reaction of ethanol on 0Pt/ZnO, 0.1Pt/ZnO, and 5Pt/ZnO NCs was studied using DRIFT spectroscopy. DRIFT peaks attributable to acetate and carbonate species were observed in 0Pt/ZnO and 0.1Pt/ZnO NCs when the NCs were exposed to air containing 50 ppm ethanol at 350 °C. These peaks were weaker in 5Pt/ZnO NCs, but hydroxyl groups were observed only in 5Pt/ZnO NCs. Gaseous products were identified by online FT-IR gas analysis. Acetaldehyde was the main product in 0Pt/ZnO and 0.1Pt/ZnO NCs, while CO_2_ was the main product in 5Pt/ZnO NCs. The results of the DRIFT and FT-IR gas analysis suggest that the partial oxidation of ethanol efficiently proceeded on ZnO with the small Pt loading (0.1Pt/ZnO NCs), leading to the largest sensor response to ethanol at 350 °C.

## Supplementary Materials

The following supporting information can be downloaded at: https://www.mdpi.com/article/10.3390/s22166277/s1, Figure S1: TEM images of Pt-ZnO NCs; Figure S2: Colloidal size distributions of Pt-ZnO NCs in toluene; Figure S3: EDS spot spectra of Pt-ZnO NCs; Figure S4: Cross-sectional SEM images of the sensor devices made of Pt-ZnO NCs; Figure S5: Temperature dependence of the sensor response; Table S1: Pt loading amounts on ZnO NCs.
