# Supplementary material for: Study on Sensing Mechanism of Volatile Organic Compounds Using Pt-Loaded ZnO Nanocrystals
Source: Sensors (Basel). 2022 Aug 20;22(16):6277. doi: 10.3390/s22166277 (PMC9415036; doi:10.3390/s22166277)
Supplement: Supplementary file 1 [file sensors-22-06277-s001.zip › sensors-1773486-supplementary.pdf]

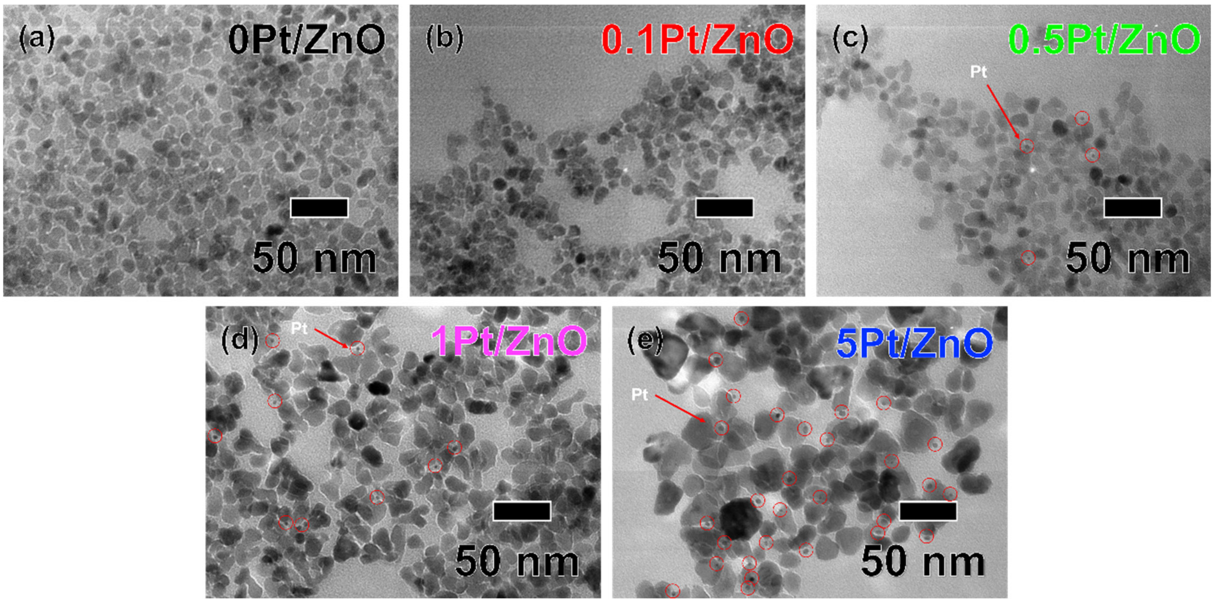

Figure S1. TEM images of (a) 0Pt/ZnO, (b) 0.1Pt/ZnO, (c) 0.5Pt/ZnO, (d) 1Pt/ZnO, and (e) 5Pt/ZnO NCs.

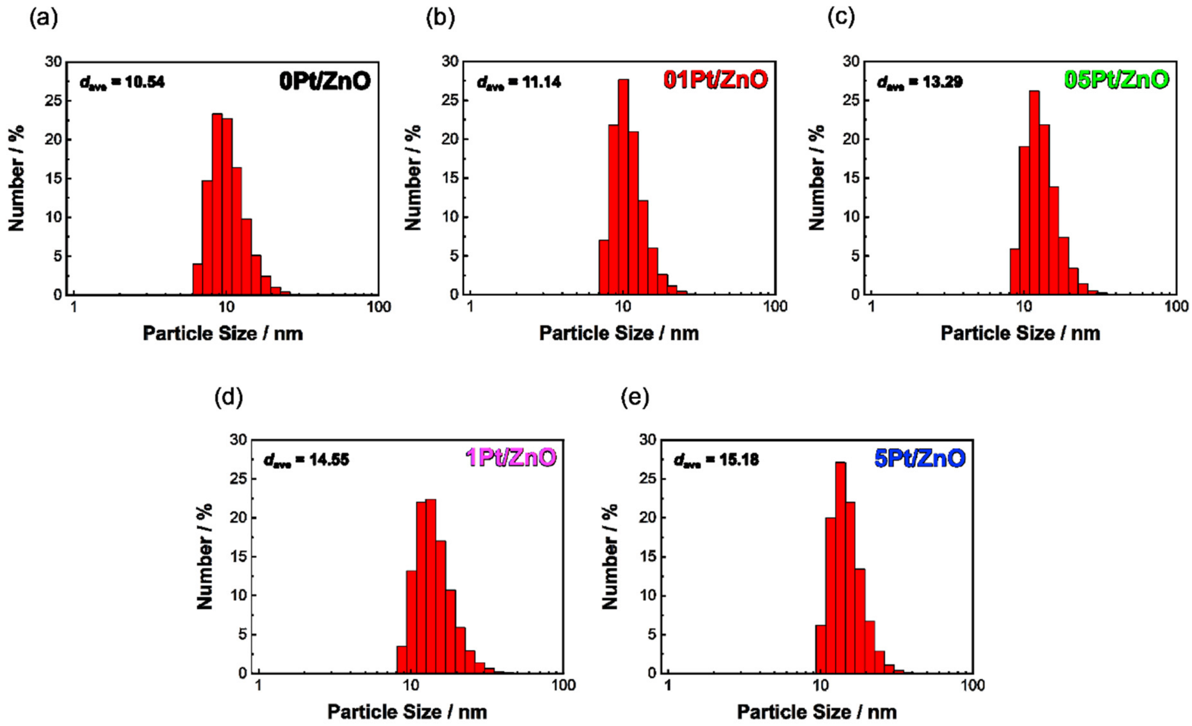

Figure S2. Colloidal size distributions of (a) 0Pt/ZnO, (b) 0.1Pt/ZnO, (c) 0.5Pt/ZnO, (d) 1Pt/ZnO, and (e) 5Pt/ZnO NCs in toluene.

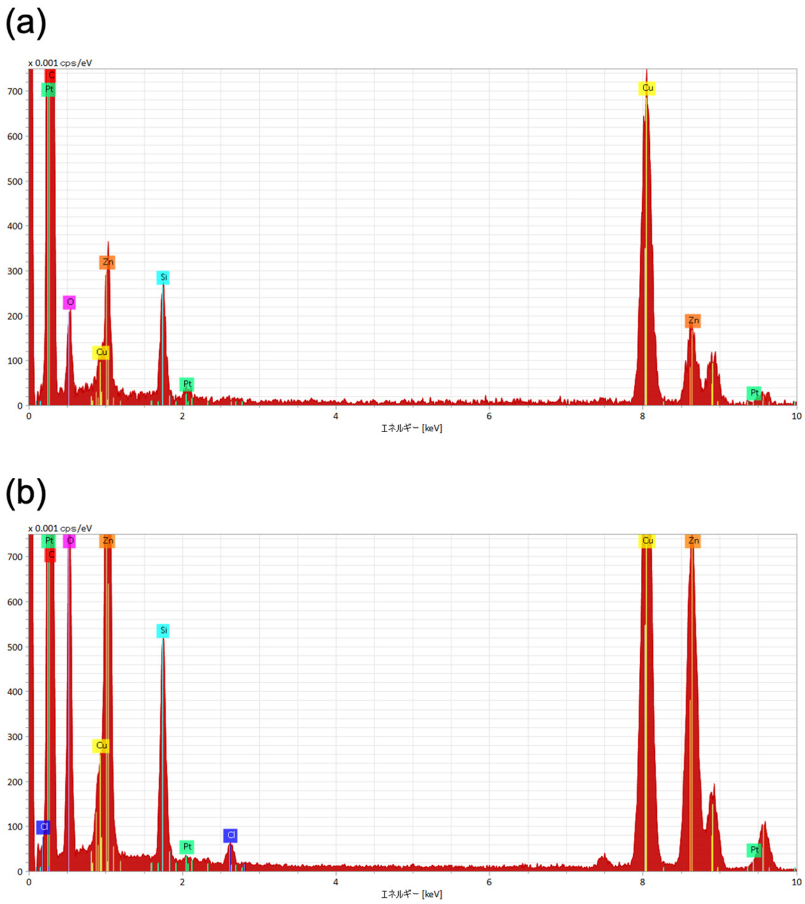

Figure S3. EDS spot spectra of (a) 0.1Pt/ZnO and (b) 5Pt/ZnO NCs.

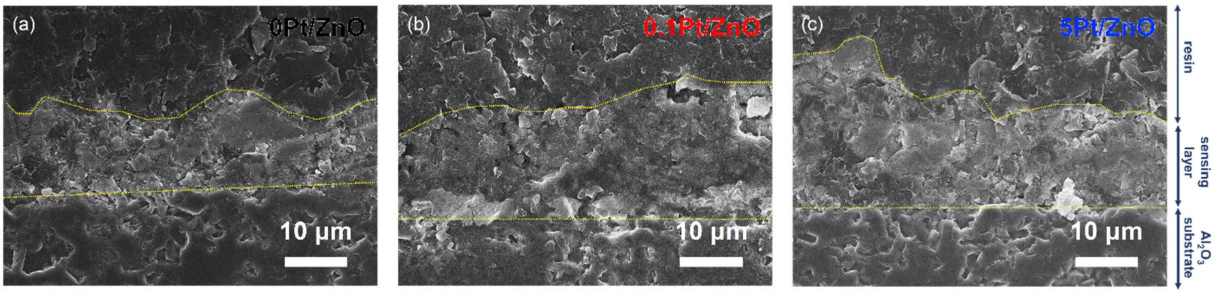

Figure S4. Cross-sectional SEM images of the sensor devices made of (a) 0Pt/ZnO, (b) 0.1Pt/ZnO, and (c) 5Pt/ZnO NCs. The thickness of the sensing layers composed of 0Pt/ZnO, 0.1Pt/ZnO, and 5Pt/ZnO is 13.5, 18.2, and 17.9 μm, respectively.

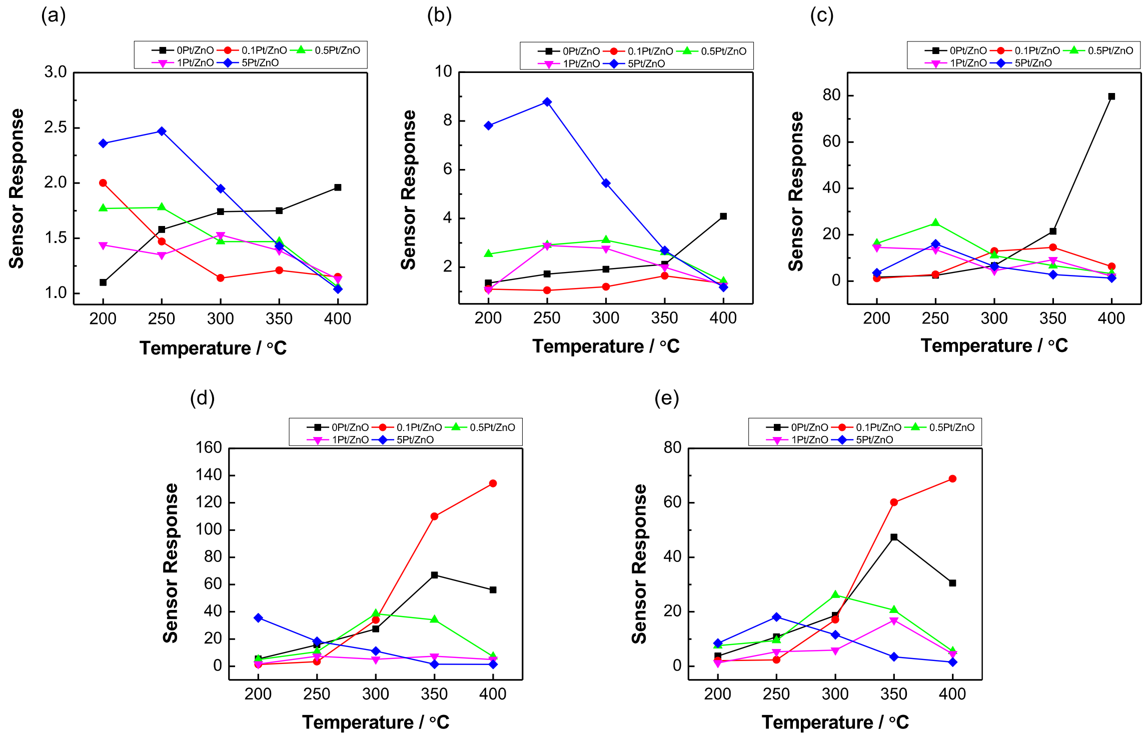

Figure S5. Temperature dependence of the sensor response to (a) CO, (b) H_2, (c) toluene, (d) acetone, and (e) ethanol for five different sensors.

Table S1. Pt loading amounts on ZnO NCs.

Sample	*Pt Loading (w%)
0.1Pt/ZnO	0.236
0.5Pt/ZnO	0.560
1Pt/ZnO	0.705
5Pt/ZnO	2.691

*The loading amounts were determined by XRF analysis.
